# Targeting the facilitative glucose transporter GLUT1 inhibits the self-renewal and tumor-initiating capacity of cancer stem cells

**DOI:** 10.18632/oncotarget.2892

**Published:** 2014-11-26

**Authors:** Keita Shibuya, Masashi Okada, Shuhei Suzuki, Manabu Seino, Shizuka Seino, Hiroyuki Takeda, Chifumi Kitanaka

**Affiliations:** ^1^ Department of Molecular Cancer Science, Yamagata University School of Medicine, Yamagata, Japan; ^2^ Oncology Research Center, Research Institute for Advanced Molecular Epidemiology, Yamagata University, Yamagata, Japan; ^3^ Global COE program for Medical Sciences, Japan Society for Promotion of Science, Tokyo, Japan; ^4^ Department of Clinical Oncology, Yamagata University School of Medicine, Yamagata, Japan; ^5^ Department of Obstetrics and Gynecology, Yamagata University School of Medicine, Yamagata, Japan; ^6^ Research Institute for Promotion of Medical Sciences, Yamagata University School of Medicine, Yamagata, Japan

**Keywords:** cancer initiating cell, glioma, tumorigenicity, xenograft analysis

## Abstract

Increased glucose metabolism is now recognized as an emerging hallmark of cancer. Recent studies have shown that glucose metabolism is even more active in cancer stem cells (CSCs), a rare population of cancer cells with the capacity to self-renew and initiate tumors, and that CSCs are dependent on glycolysis for their survival/growth. However, the role of glucose metabolism in the control of their self-renewal and tumor-initiating capacity per se still remains obscure. Moreover, much remains unknown as to which of the numerous molecules involved in the glucose metabolism is suitable as a target to control CSCs. Here we demonstrate that the facilitative glucose transporter GLUT1 is essential for the maintenance of pancreatic, ovarian, and glioblastoma CSCs. Notably, we found that WZB117, a specific GLUT1 inhibitor, could inhibit the self-renewal and tumor-initiating capacity of the CSCs without compromising their proliferative potential *in vitro*. *In vivo*, systemic WZB117 administration inhibited tumor initiation after implantation of CSCs without causing significant adverse events in host animals. Our findings indicate GLUT1-dependent glucose metabolism has a pivotal role not only in the growth and survival of CSCs but also in the maintenance of their stemness and suggest GLUT1 as a promising target for CSC-directed cancer therapy.

## INTRODUCTION

Cancer stem cells (CSCs) are a small subpopulation of cancer cells having the capacity to self-renew as well as the capacity to initiate and recapitulate tumors from which they are derived [1, 2]. Compared to non-stem cancer cells that account for the majority of tumor cells comprising a tumor, CSCs are often refractory to conventional chemo- and radiotherapies and can survive even treatments that would eradicate non-stem cancer cells, causing tumor recurrence after seemingly successful cancer treatments [3-6]. As such, CSCs are now deemed as ringleaders that perpetrate tumor recurrence, and elimination of CSCs, either functional (inducing differentiation of CSCs into non-stem cancer cells) or physical (inducing cell death of CSCs), is therefore expected to be key to curative treatment of cancer [7, 8].

It has long been recognized that increased glucose uptake associated with increased glycolysis is a characteristic feature of cancer cells, and this propensity of cancer cells to actively take up and metabolize glucose has formed the basis for fluorodeoxyglucose imaging as a highly useful cancer detection method [9-11]. The role and significance of increased glucose metabolism in cancer cell biology, therefore, has been a focus of intense research. To date, accumulating evidence suggests that such alterations in the glucose metabolism of cancer cells may contribute to their proliferation, survival, metastasis, and therapy resistance [12-16]. However, since studies have been conducted essentially using non-stem cancer cells, the role of glucose metabolism in the control of CSCs has been scarcely investigated and thus remains largely unknown.

Recently, we discovered that lowering the extracellular glucose concentration remarkably enhances the ability of metformin, an anti-diabetic agent, to differentiate glioma stem cells (GSCs) into non-stem glioma cells [17], which suggested the possibility that cellular glucose metabolism driven by concentration-dependent glucose uptake may have a role in the maintenance of the stem cell status of GSCs. Given that glucose is transported via two classes of hexose transporters, namely, the SGLT (sodium-dependent glucose transporter) family and the GLUT (facilitative glucose transporter) family, and that SGLTs transport glucose against the concentration gradient whereas GLUTs do so along the gradient [18], our earlier observation may suggest that GLUTs are involved in the control of GSCs.

Here in this study, we therefore explored the possibility that GLUTs are involved in the control of CSCs from different cancer types including GSCs. Among the 14 members of mammalian GLUTs, we focused on GLUT1, which is reportedly expressed abundantly in cancer cells [18-21]. Our results indicate that GLUT1 expression and activity is essential for the maintenance of the self-renewal and tumor-initiating capacity of CSCs, suggesting that CSC maintenance may be among the critical roles of increased glucose metabolism of cancer cells and that GLUT1 could be a promising molecular target in CSC-directed cancer therapies.

## RESULTS

### The impact of glucose concentration in the culture medium on CSCs

**Figure 1:**
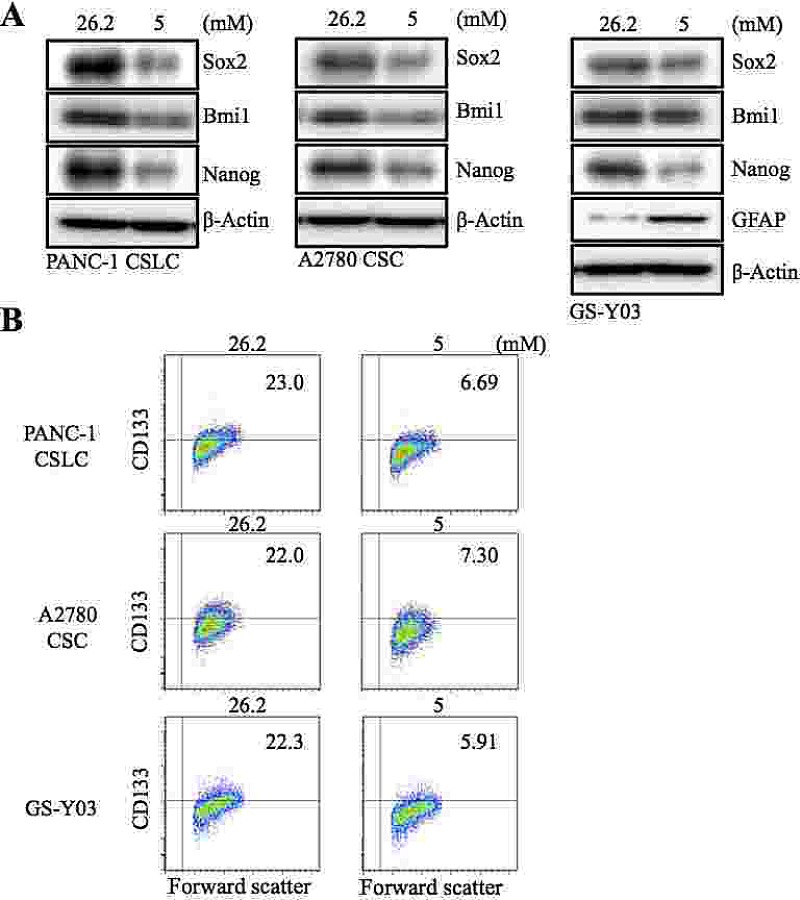
The effect of glucose concentration in the culture medium on the stem cell and differentiation marker expression of CSCs The indicated cancer stem cells (PANC-1 CSLC, A2780 CSC, and GS-Y03) were cultured for 7 days in the stem cell culture media containing either 26.2 mM or 5 mM glucose. The cells were then examined for the expression of the indicated stem cell and differentiation markers by immunoblot analysis (A) and for the cell surface expression of CD133 by flow cytometry (B). In (B), representative flow cytometric plots are shown together with the percentages of CD133-positive cells.

In our earlier study, we showed that decreasing the glucose concentration in the culture medium from 26.2 mM to 17.5 mM remarkably sensitized GSCs to the differentiation-inducing effect of metformin [17]. To determine, in this study, the impact of lowering the glucose concentration in the absence of metformin further down to the physiological range, we cultured human CSCs from different cancer types (PANC-1 CSLC, pancreatic cancer; A2780 CSC, ovarian cancer; GS-Y03, glioblastoma) in the presence of 5 mM glucose, a concentration which falls within the normal blood glucose range. When these CSCs were examined for their stem cell marker expression after being cultured in the low-glucose medium for 1 week, we found that the expression of stem cell markers such as Sox2, Bmi1, and Nanog (Figure 1 A), as well as the surface expression of CD133 (Figure 1 B), was reduced in all 3 types of CSCs. To determine whether the loss of the stem cell marker expression actually reflected loss of stemness associated with differentiation, we examined the expression of glial fibrillary acidic protein (GFAP), an astrocytic differentiation marker, in GS-Y03. GFAP expression was induced when GS-Y03 cells were cultured in the presence of 5 mM glucose for 1 week, which suggested that the cells had undergone astrocytic differentiation in the low-glucose culture condition (Figure 1 A). Thus, the results suggested that, at least in the *in vitro* stem cell culture condition used in this study, CSCs may not be able to maintain their stem cell state at a physiological glucose concentration and may therefore require supra-physiological concentrations of glucose for their maintenance.

### Pharmacological inhibition of GLUT1 inhibits the self-renewal capacity of CSCs

**Figure 2:**
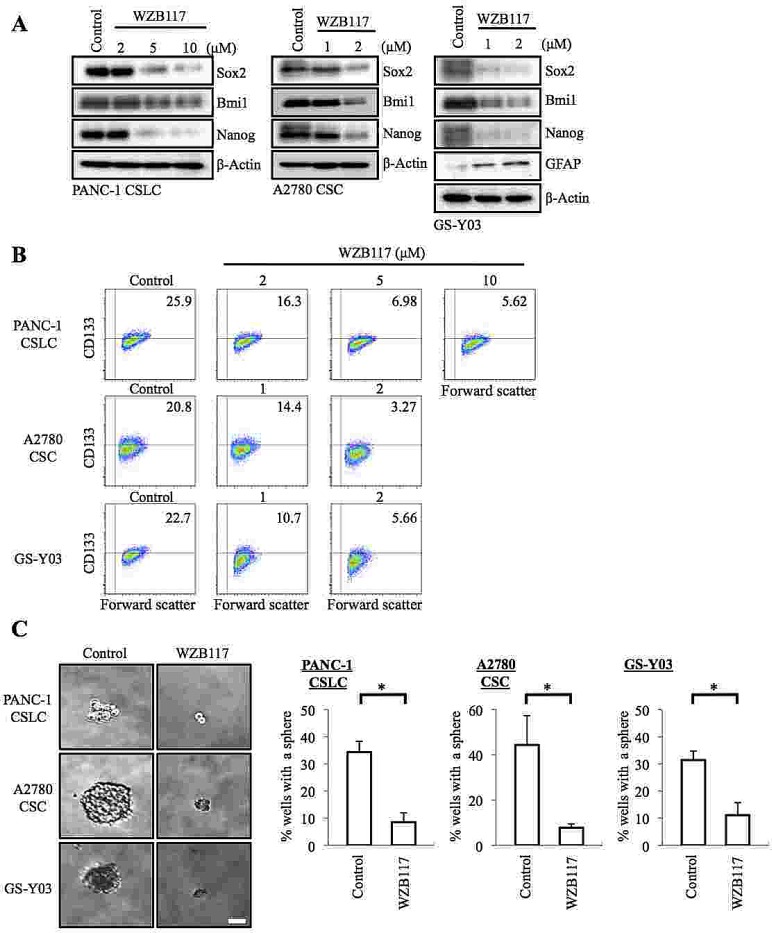
Pharmacological inhibition of GLUT1 by WZB117 causes loss of the self-renewal capacity of CSCs (A, B) Cells cultured in the absence (Control) or presence of WZB117 as indicated for 6 days were subjected to immunoblot analysis of the indicated proteins (A) and to flow cytometric analysis for the cell surface expression of CD133 (B). In (B), representative flow cytometric plots are shown together with the percentages of CD133-positive cells. (C) Cells were cultured in the absence (Control) or presence of WZB117 (10 μM for PANC-1 CSLC and 2 μM for A2780 CSC and GS-Y03) for 6 days and subjected, after washout of the inhibitor, to the sphere formation assay in the absence of WZB117. Left panels show the photographs of representative wells. The graphs show the percentage of wells in which a tumor sphere was formed from a single cell. Values represent means + SD from three independent experiments. *p<0.01. Bar: 50 μm.

Since the CSCs examined in this study were thus highly dependent on the extracellular glucose concentration for their maintenance, we surmised that glucose metabolism may have a role in the maintenance of their self-renewal capacity and that facilitative glucose transporters (GLUTs), which facilitate the energy-independent diffusion of glucose across the cell membrane down the glucose concentration gradient, may be involved in their glucose uptake, which is a prerequisite for the initiation of intracellular glucose metabolism. To test these ideas and examine the possible role of GLUT1 in CSCs, we first took advantage of a specific pharmacological inhibitor of GLUT1, WZB117 [22]. Because we wished to examine the effect of WZB117 specifically on the self-renewal capacity (i.e., not on the viability) of CSCs, we determined the non-toxic concentrations of WZB117 for each CSC. We then treated PANC-1 CSLCs, A2780 CSCs, and GS-Y03 cells with WZB117 at their respective non-toxic concentrations for 6 days, after which the cells were examined for stem cell and differentiation marker expression. The results indicated that WZB117 inhibited and induced the expression of the stem cell and differentiation markers, respectively, in a concentration-dependent manner in the CSCs examined (Figure 2 A, B). Under the experimental conditions used in this study, we confirmed that the glucose uptake of the CSCs was reduced by the WZB117 treatment (Supplemental Figure S1) and that their glucose metabolism was accordingly inhibited as suggested by the activation of a cellular energy sensor AMPK upon WZB117 treatment (Supplemental Figure S2).

Since the results suggested that sublethal impairment of glucose metabolism could inhibit the self-renewal capacity of the CSCs, we next examined whether WZB117 inhibits the sphere-forming ability of the CSCs without compromising their viability. The results of the sphere formation assay demonstrated that 6-day pretreatment with WZB117 was sufficient to deprive the CSCs of their ability to form spheres even in the absence of WZB117 (Figure 2 C). Most importantly, when the CSCs were cultured under the monolayer stem cell culture condition after the identical 6-day WZB117 pretreatment, we observed no significant differences in growth (i.e., the numbers of viable and dead cells) between the control-treated and WZB117-treated CSCs (Supplemental Figure S3). The results imply that WZB117 specifically targets the sphere-forming ability of the CSCs without impairing their proliferative potential per se.

### Genetic knockdown of GLUT1 inhibits the self-renewal capacity of CSCs

**Figure 3:**
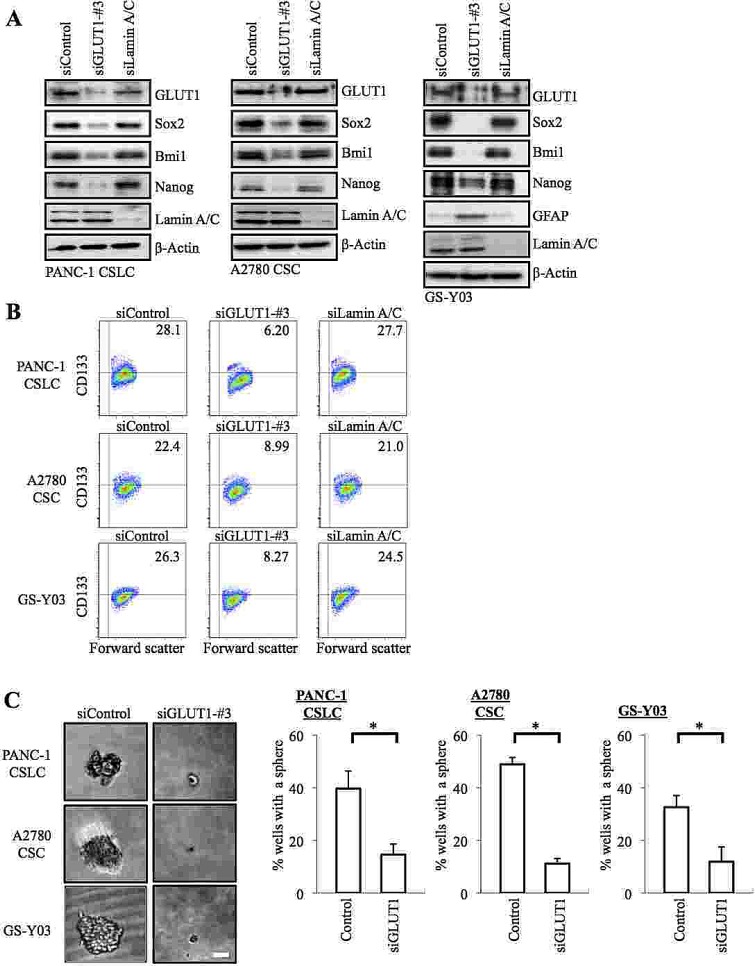
Gene silencing of GLUT1 by siRNA causes loss of the self-renewal capacity of CSCs (A) Cells were transiently transfected with an siRNA against GLUT1 (siGLUT1-#3), and as controls, with an siRNA against Lamin A/C (siLamin A/C) or with a non-targeting siRNA (siControl), as detailed in Materials and methods. The transfected cells were then subjected, 6 days after transfection for PANC-1 CSLCs and 8 days after transfection for A2780 CSCs and GS-Y03 cells, to immunoblot analyses for the expression of the indicated proteins. (B) Cells treated as in (A) were subjected to flow cytometric analysis for the cell surface expression of CD133. Representative flow cytometric plots together with the percentages of CD133-positive cells are shown. (C) Cells treated as in (A) were subjected to the sphere formation assay. Left panels show the photographs of representative wells. The graphs show the percentage of wells in which a tumor sphere was formed from a single cell. Values represent means + SD from three independent experiments. *p<0.01. Bar: 50 μm.

The results of the inhibitor experiments suggested that GLUT1 activity was required for the glucose uptake as well as for the maintenance of the self-renewal capacity of the CSCs. To definitively determine the role of GLUT1 in the CSCs, we next conducted GLUT1 knockdown experiments. Introduction of an siRNA against GLUT1, but not a non-targeting siRNA or an siRNA against lamin A/C, resulted in decreased GLUT1 expression (Figure 3A) and glucose uptake (Supplemental Figure S5) of the CSCs. Under the experimental condition, we confirmed that the stem cell marker expression was decreased, whereas the differentiation marker expression was induced (Figure 3 A, B), in GLUT1 knockdown cells compared to the control cells, which was reproduced using another siRNA against GLUT1 (Supplemental Figure S4). In the sphere formation assay, GLUT1 knockdown cells formed spheres significantly less efficiently than the control cells (Figure 3 C). These results, in conjunction with those of the pharmacological inhibitor experiments, suggest that GLUT1 plays an essential role in the maintenance of the glucose metabolism and self-renewal capacity of the CSCs.

### Targeting GLUT1, either *in vitro* or *in vivo*, effectively deprives CSCs of their tumor-initiating capacity

**Figure 4:**
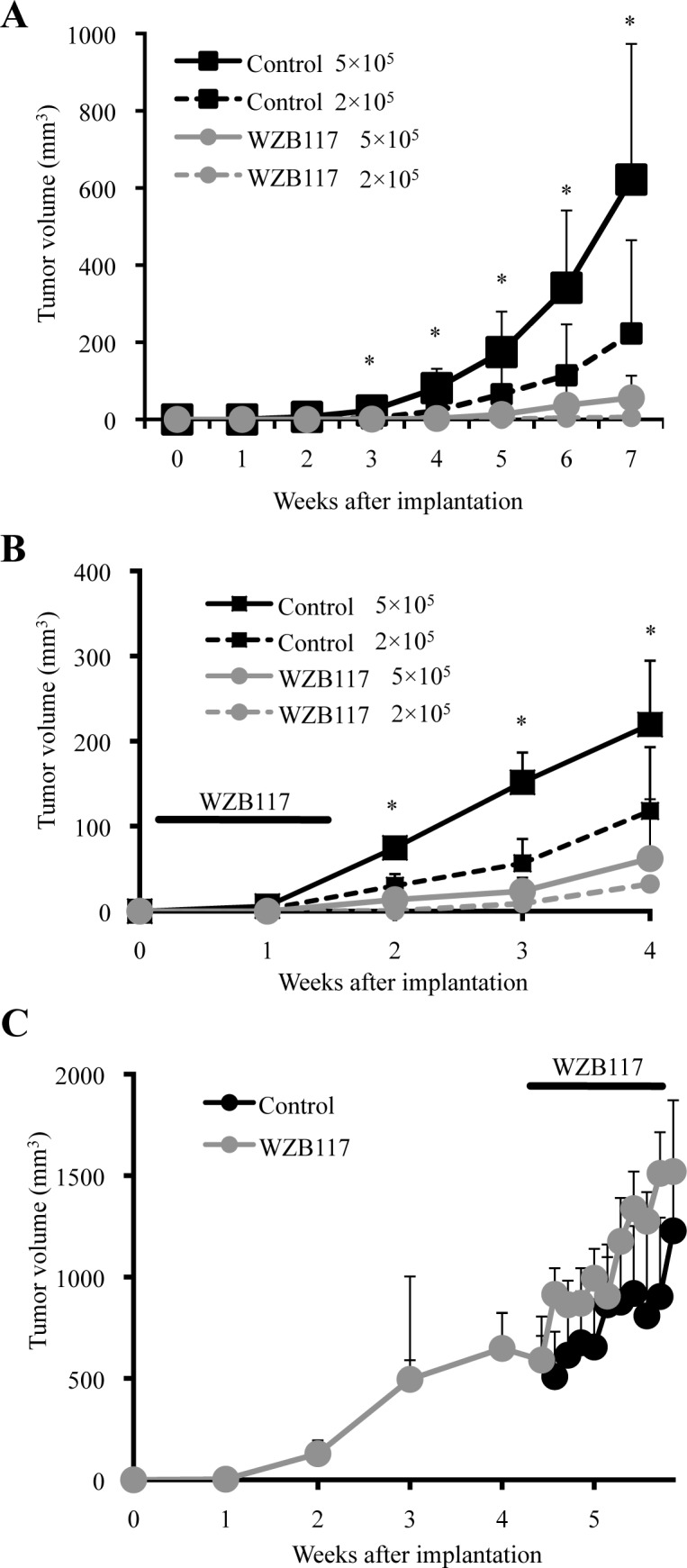
Targeting GLUT1, either *in vitro* or *in vivo*, effectively inhibits tumor initiation by CSCs (A) Mice (3 for each group) were implanted subcutaneously with the indicated numbers of viable PANC-1 CSLCs that had been treated without (Control) or with 10 μM WZB117 for 6 days. (B) Mice implanted subcutaneously with the indicated numbers of viable PANC-1 CSLCs (3 for each group) were administered a daily intraperitoneal injection of the control vehicle or WZB117 (20 mg/kg/day) for 10 consecutive days starting on the next day of implantation.(C) Mice (4 for each group) were implanted subcutaneously with PANC-1 CSLCs. After 30 days, when the tumor volumes ranged from 292 to 1080 mm^3^, the mice were randomized and received a daily intraperitoneal injection of the control vehicle or WZB117 (20 mg/kg/day) for 10 consecutive days. In (A) through (C), tumor volume was measured at the indicated time points after implantation, and the results are presented in the graphs as means + SD of each group. *p<0.05 (WZB117 5 × 10^5^ compared against Control 5 × 10^5^ in (A) and (B)).

Along with self-renewal capacity, tumor-initiating capacity is another key essential property intrinsic to CSCs. Given the critical role of GLUT1 in the maintenance of the self-renewal capacity of the CSCs, we next asked if we could inhibit, similarly to the self-renewal capacity, the tumor-initiating capacity of the CSCs by targeting GLUT1 *in vitro*, and from a therapeutic point of view, *in vivo* as well. To rigorously test this point, we chose for the following experiments PANC-1 CSLCs, which was the most resistant to GLUT1 inhibition among the three CSCs, i.e., requiring the highest concentration of WZB117 for inhibition of self-renewal capacity and cell viability. To determine first whether targeting GLUT1 with WZB117 inhibits the tumor-initiating capacity of PANC-1 CSLCs, we examined, after pretreatment with WZB117 *in vitro*, their ability to initiate tumors upon xenotransplantation into nude mice. PANC-1 CSLCs were treated with WZB117 at 10 μM for 6 days, a condition which did not significantly inhibit their viability and monolayer growth after washout of the inhibitor (Supplemental Figure S3). When the cells were then implanted into nude mice, tumor formation by the WZB117-treated cells was significantly inhibited compared to the control-treated cells, indicating that WZB117 can selectively target the tumor-initiating capacity of PANC-1 CSLCs *in vitro* without compromising their viability and proliferative potential (Figure 4 A). We then went on to ask whether WZB117 could inhibit tumor initiation by PANC-1 CSLCs *in vivo*. To address this question, we implanted PANC-1 CSLCs into nude mice, and on the next day of implantation, started systemic administration of WZB117 for 10 consecutive days, after which the mice were monitored without further administration of the drug. The results indicated that WZB 117 inhibited the tumor-initiating capacity of PANC-1 CSLCs also *in vivo*, with the inhibitory effect of systemic WZB117 administration on tumor initiation being more pronounced than that of reducing the number of implanted cells by 2.5-fold (Figure 4 B). We also confirmed that the protocol of WZB117 treatment used in this study (20 mg/kg/day for 10 consecutive days) was well tolerated by nude mice and did not affect their general health status for at least 5 months (Supplemental Figure S6). Finally, to determine whether, *in vivo*, WZB117 inhibits tumor initiation selectively or tumor growth in general, we examined the effect of systemic administration of WZB117 on pre-established tumors. We implanted PANC-1 CSLCs into nude mice and allowed them to grow to form tumors. When the tumors reached the size of ~ 600 mm^3^, we started systemic administration of WZB117 to the host mice with exactly the same protocol that effectively inhibited tumor initiation by PANC-1 CSLCs as described above. Strikingly, the protocol had no discernible inhibitory effect on the growth of the pre-established tumors, in support of the idea that WZB117 selectively targets CSCs at least in our experimental conditions (Figure 4 C).

## DISCUSSION

In this study, we investigated the possible involvement of GLUT1, a member of the facilitative glucose transporter (GLUT) family, in the control of the self-renewal and tumor-initiating capacity of CSCs. Our results showed that both pharmacological and genetic inhibition of GLUT1 in CSCs could cause decreased stem cell marker expression, induction of differentiation marker expression, and loss of sphere-forming ability without compromising the viability of CSCs, which was confirmed in CSCs from three different types of human cancer. Consistent with such an essential role of GLUT1 in the maintenance of self-renewal capacity of CSCs, transient inhibition of GLUT1 prevented CSCs from initiating tumors, underscoring its role in the maintenance of the tumor-initiating capacity of CSCs. Thus, the results suggest that GLUT1 may play a key role in the maintenance of the self-renewal and tumor-initiating capacity of CSCs. Although we cannot formally exclude the possibility that GLUT1 contributes to the maintenance of CSCs through an as yet unknown function(s) other than glucose transport, our results might also imply that the increased glycolytic metabolism characteristically seen in human cancers has a pivotal role in the maintenance of CSCs in different, albeit not all, human cancer types. Importantly, inhibition of GLUT1 *in vivo* through systemic administration of WZB117 inhibited tumor initiation by CSCs, suggesting that GLUT1 may be a promising candidate as a target for CSC-directed cancer therapy. Notably, although the same protocol of systemic WZB117 administration failed to inhibit the growth of pre-established tumors, we meanwhile found that the proportion of CD133-positive cells in WZB117-treated tumors was smaller compared to control treated tumors (Supplemental Figure S7). Together, these results appear to suggest that, *in vivo*, CSCs might be more sensitive to GLUT1 inhibition than non-stem cancer cells and that WZB117 may selectively target CSCs at least in our experimental condition.

It has long been recognized that cancer cells in general show increased glycolytic activity, and recent evidence suggests that CSCs may have even more increased glycolytic activity compared to non-stem cancer cells [10, 23-26]. However, the role and significance of this propensity of CSCs toward highly glycolytic metabolism remain largely unknown. Recent studies have shown that 3-bromopyruvate, a hexokinase 2 (HK2) inhibitor, and its derivatives (pentyl 3-bromopyruvate ester [P-BrPE] and 3-bromo-2-oxopropionate-1-propyl ester [3-BrOP]) reduce the viability of CSCs and that knockdown of GLUT3 inhibits the growth of brain tumor CSCs [23, 25-28]. While all these studies do indicate that the glycolytic metabolism plays an essential role in the survival and proliferation of CSCs, it still remains to be shown whether the glycolytic metabolism has a role in the maintenance of the self-renewal capacity of CSCs. Here in the present study, we have provided for the first time evidence to support the idea that the active glycolytic metabolism characteristic of CSCs plays an essential role not only in their survival and proliferation but also in the maintenance of the self-renewal and tumor-initiating capacity of CSCs. Given the role of glycolysis as a critical cellular process required for energy production and macromolecular synthesis [10, 29], it is quite natural that inhibition of glycolysis leads to reduced cellular viability and proliferation. On the other hand, the mechanism by which glycolysis contributes to the maintenance of CSC self-renewal remains rather enigmatic. One intriguing possibility is that glycolysis has a role in the regulation of redox status of CSCs [30], which, as we have recently demonstrated, is a key determinant of CSC fate [31]. Whether or not glycolysis controls the stemness of CSCs via redox regulation, the mechanism by which glycolysis controls the stemness of CSCs will be an important issue of future investigation.

The findings of this study are of substantial significance also from a therapeutic perspective. One of the most critical problems inherently associated with targeting glycolysis for cancer therapy is that glycolysis is commonly utilized by cells in general as a major source of energy and macromolecule supply irrespective of whether they are neoplastic or not [10, 29]. So, it is highly desired that, when targeting glycolysis for cancer therapy, we find out such target molecules that cancer cells specifically require to maintain their functional integrity. In this regard, GLUT1 is an attractive candidate as a target molecule, since GLUT1 is expressed preferentially in cancer cells [18-21]. Importantly, the concentrations of WZB117 required for the inhibition of the self-renewal and tumor-initiating capacity of the CSCs were apparently lower than those required to reduce their viability, which will help create an even larger therapeutic window. Indeed, in the xenograft analyses of the present study, tumor initiation by PANC-1 CSLCs was successfully inhibited by systemic WZB117 treatment, which neither caused adverse events nor inhibited bulk tumor growth. Thus, the results clearly indicate that GLUT1 is a promising molecular target for CSC-directed cancer therapy. Similarly to GLUT1, GLUT3 is another promising target to control the glycolytic metabolism of CSCs [27]. To date, the effects of specific GLUT3 inhibitors on CSCs and on normal cells or individuals have yet to be reported and therefore remain unknown. However, given the possible role of GLUT3 in various human cancer types [27], GLUT3 may remain a strong candidate as a target for CSC-directed therapy. HK2 is another molecule of interest in this regard, since HK2 has been shown to be specifically required for tumor initiation and maintenance in mouse models of cancer while being dispensable in adult mice without causing an overt phenotype [32]. In accordance with such a report, BrPE and BrOP, though they may not necessarily be specific inhibitors of HK2 [33], have been shown to reduce the viability of CSCs *in vitro* [23, 25, 26]. *In vivo*, the cancer therapeutic effects of these inhibitors have been demonstrated alone or in combination with conventional chemotherapeutic agents [23, 25, 26]. Because of the limited number of relevant studies, it still remains to be determined which molecule(s) in the glycolytic pathway is superior to another as a therapeutic target. Nevertheless, all relevant studies including ours at least unanimously indicate that the glycolytic pathway is a viable target in CSC-directed cancer therapy.

In conclusion, we have provided evidence that the active glucose transport of CSCs has a role in the maintenance of their self-renewal and tumor-initiating capacity and is therefore an attractive target of CSC-directed therapy. We have also demonstrated that GLUT1 is a promising molecular target to specifically control CSCs *in vivo*. In combination with other therapeutic modalities that effectively control the tumor bulk, glucose metabolism-targeted therapy through GLUT1 inhibition is expected to help achieve better management of cancers through prevention of fatal recurrence.

## MATERIALS AND METHODS

### Antibodies and reagents

Anti-Sox2 (#3579), anti-Nanog (#4903), anti-Bmi1 (#6964), anti-GFAP (#3670), phospho-AMPKα (#2535), AMPKα (#2603) antibodies were purchased from Cell Signaling Technology, Inc. (Beverly, MA, USA). Anti-β-actin (A1978) was from Sigma (St. Luis, MO, USA). Anti-GLUT1 antibody (sc-7903) was from Santa Cruz Biotechnology, Inc. (Santa Cruz, CA, USA). Anti-CD133 (W6B3C1) was from Miltenyi Biotech (Germany). Anti-Lamin A/C (ab8984) was purchased from Abcam (Cambridge, UK). WZB117 (SML0621) was from Sigma.

### Cell culture

The establishment of the human CSCs used in this study (GS-Y03, PANC-1 CSLC, and A2780 CSC) has been described [17, 34, 35]. These cell lines were maintained under the monolayer stem cell culture condition reported previously [17, 34, 35]. In brief, cells were cultured on collagen-I-coated dishes (IWAKI, Tokyo, Japan) in the stem cell culture medium (DMEM/F12 medium supplemented with 1% B27 [Gibco-BRL, Carlsbad, CA, USA], 20 ng/mL EGF and FGF2 [Peprotech, Inc., Rocky Hill, NJ, USA], D-(+)-glucose [final concentration, 26.2 mM or 5 mM where indicated as such], L-glutamine [final concentration, 4.5 mM], 100 units/mL penicillin and 100 μg/mL streptomycin). In principle, the stem cell culture medium was changed every 3 days, and EGF and FGF2 were added to the culture medium every day. Throughout the study, the cell number was determined using a hemocytometer, and the cell viability was examined by the dye exclusion method (0.2% trypan blue). Cell viability (%) was defined as 100 x ‘the number of viable cells’/(‘the number of viable cells’ + ‘the number of dead cells’).

### Gene silencing by siRNA

siRNAs against human GLUT1 (SLC2A3; #1: HSS109811, #3: HSS185757) and Medium GC Duplex #2 of Stealth RNAi^TM^ siRNA Negative Control Duplexes (as a non-targeting control for siRNA experiments) were purchased from Invitrogen Life Technologies (Carlsbad, CA, USA). An siRNA against human Lamin A/C (siGENOME^TM^ Control siRNA, D-001050-01-20) was purchased from Thermo Fisher Scientific Inc. (Waltham, MA, USA). Transfection of siRNAs was performed using Lipofectamine RNAiMAX (Life Technologies) according to the manufacturer's instructions. To achieve sustained knockdown of the target genes, siRNA transfection was repeated 3 days (for PANC-1 CSLCs) or 4 days (for A2780 CSCs and GS-Y03 cells) after the initial transfection.

### Sphere formation assay

After being dissociated into single cells, PANC-1 CSLCs, A2780 CSCs, and GS-Y03 cells were serially diluted in the stem cell culture medium and seeded into non-coated 96-well plates so that there be a single cell in each well. Wells containing a single cell were marked on the next day and, 1 week after seeding, the percentage of marked wells with a sphere relative to the total number of marked wells was determined.

### Flow cytometric analysis

Dissociated cells were washed with ice-cold phosphate-buffered saline (PBS), fixed with 4% (w/v) paraformaldehyde for 10 min at room temperature (RT), and washed again with PBS. The cells were then blocked in FACS buffer (0.5% [w/v] bovine serum albumin, 0.1% [w/v] NaN_3_ in PBS) for 30 min, followed by 3 PBS rinses and subsequently by incubation with the anti-CD133 antibody in the FACS buffer for 1 h and then with the Alexa Fluor^®^ 488 goat anti-mouse IgG for another 30 min at RT. Gating for single cells was established using forward scatter in the isotype control samples. The isotype control samples were used to establish a gate in the fluorescein isothiocyanate (FITC) channel. Cells showing signal for CD133 above the gate established by the isotype control were deemed CD133-positive. All flow cytometric experiments were run on FACSCanto^TM^ II Flow Cytometer (BD Biosciences, Franklin Lakes, NJ, USA) and the data were analyzed using FlowJo software, version 7.6.5 (Treestar Inc., Ashland, OR, USA).

### Immunoblot analysis

Cells were washed with ice-cold PBS and lysed in RIPA buffer (10 mM Tris-HCl [pH 7.4], 0.1% SDS, 0.1% sodium deoxycholate, 1% NP-40, 150 mM NaCl, 1 mM EDTA, 1.5 mM Na_3_VO_4_, 10 mM NaF, 10 mM sodium pyrophosphate, 10 mM sodium β-glycerophosphate and 1% protease inhibitor cocktail set III [Calbiochem]). After centrifugation for 10 min at 14,000 x g at 4 °C, the supernatants were recovered as the cell lysates, and the protein concentration of the cell lysates was determined by the BCA protein assay kit (Pierce Biotechnology, Inc., Rockford, IL, USA). Cell lysates containing equal amounts of protein were separated by SDS-PAGE and transferred to a polyvinylidene difluoride membrane. The membrane was probed with a primary antibody and then with an appropriate HRP-conjugated secondary antibody according to the protocol recommended by the manufacturer of each antibody. Immunoreactive bands were visualized using Immobilon Western Chemiluminescent HRP Substrate (Millipore, Billerica, MA, USA).

### Glucose-uptake assay

The glucose-uptake assay was done essentially as described [36]. In brief, cells were treated with 1 mM 2-deoxyglucose (2DG) for 20 min at 37°C 5% CO_2_. The reaction was stopped by harvesting the cells and washing them with ice-cold PBS three times. The cell pellet was solubilized in 10 mM Tris-HCl (pH7.4) by sonication (UH-50, SMT CO., LTD, Tokyo, JAPAN), followed by determination of the amount of 2DG using 2DG Uptake Measurement Kit (OKP-PMG-K01T, COSMO BIO Co., LTD, Tokyo, JAPAN), according to the manufacturer's instructions. Glucose uptake (pg/min/cell) was calculated as the amount of 2DG (pg) transported per cell per minute.

### Mouse studies

Mouse xenograft studies were carried out essentially as previously described [34]. For subcutaneous implantation, 5- to 8-week-old male BALB/cAJcl-nu/nu mice (Clea Japan, Inc.) were, after being anesthetized with avertin (0.375 g/kg intraperitoneally), implanted subcutaneously in the flank region with cells suspended in 200 μL of PBS. After implantation, the recipient mice were monitored for general health status and the presence of subcutaneous tumors. Tumor volume was determined by measuring tumor diameters (measurement of 2 perpendicular axes of tumors) using a caliper and calculated as 1/2 x (larger diameter) x (smaller diameter)^2^. For systemic administration of WZB117, the WZB117 stock solution (4 mg/mL in dimethylsulfoxide [DMSO]) was diluted in PBS to prepare 200 μL solutions of WZB117 for each injection. The WZB117 solutions were injected intraperitoneally into nude mice. Note that all the control- and WZB117-treated mice received an equal volume of DMSO per body weight (3.6 mL/kg/day). For flow cytometric analysis of tumor cells, subcutaneous tumors were excised and, after being washed in chilled sterile PBS, transferred into DMEM/F12 containing penicillin and streptomycin. The excised tumors were then minced with scissors and incubated in a dissociation enzyme solution (20 mM HEPES-PBS [pH7.4] containing 0.02% DNase I [Sigma, A4572] and 0.02% collagenase II [Sigma, C6885]) for 30 min at 37 °C. After being rinsed with DMEM/F12, dissociated tumor cells were re-suspended in DMEM/F12 and filtered through a 70-μm strainer. The dissociated cells were then fixed and subjected to flow cytometric analysis. All animal experiments were performed under a protocol approved by the Animal Research Committee of Yamagata University.

### Statistical analysis

Results are expressed as means and standard deviation (SD), and differences were compared using the 2-tailed Student's *t*-test. P-values < 0.05 were considered statistically significant and indicated with asterisks in the figures.

## SUPPLEMENTARY MATERIAL AND FIGURES


