# Supplementary material for: Targeting the facilitative glucose transporter GLUT1 inhibits the self-renewal and tumor-initiating capacity of cancer stem cells
Source: Oncotarget. 2014 Nov 26;6(2):651–61. doi: 10.18632/oncotarget.2892 (PMC4359246; doi:10.18632/oncotarget.2892)
Supplement: Supplementary file 1 [file oncotarget-06-651-s001.pdf]

Targeting the facilitative glucose transporter GLUT1 inhibits the self-renewal and tumor-initiating capacity of cancer stem cells

Supplementary Material

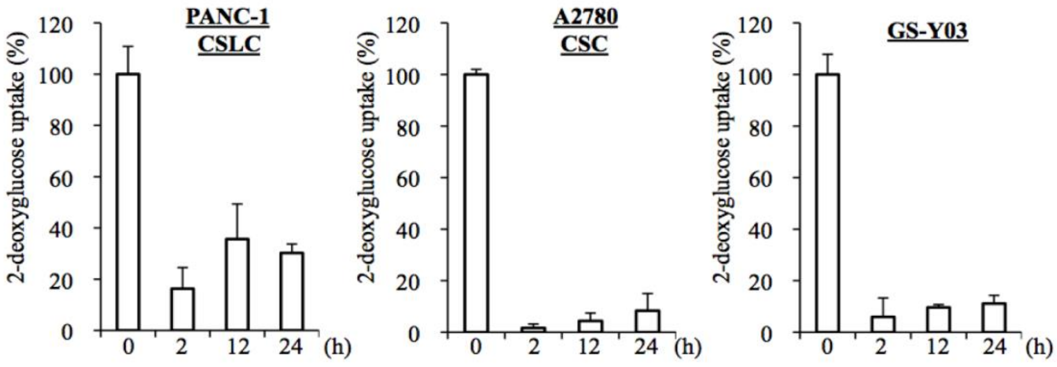

Supplemental Figure S1: The effect of WZB117 treatment on glucose uptake of CSCs.

Cells cultured in the presence of WZB117 (10 μM for PANC-1 CSLC and 2 μM for A2780 CSC and GS-Y03) were subjected to glucose-uptake assay at the indicated time points. Values are expressed in percentage relative to those of 0 h (mean values set to 100) and represent means + SD of three independent experiments.

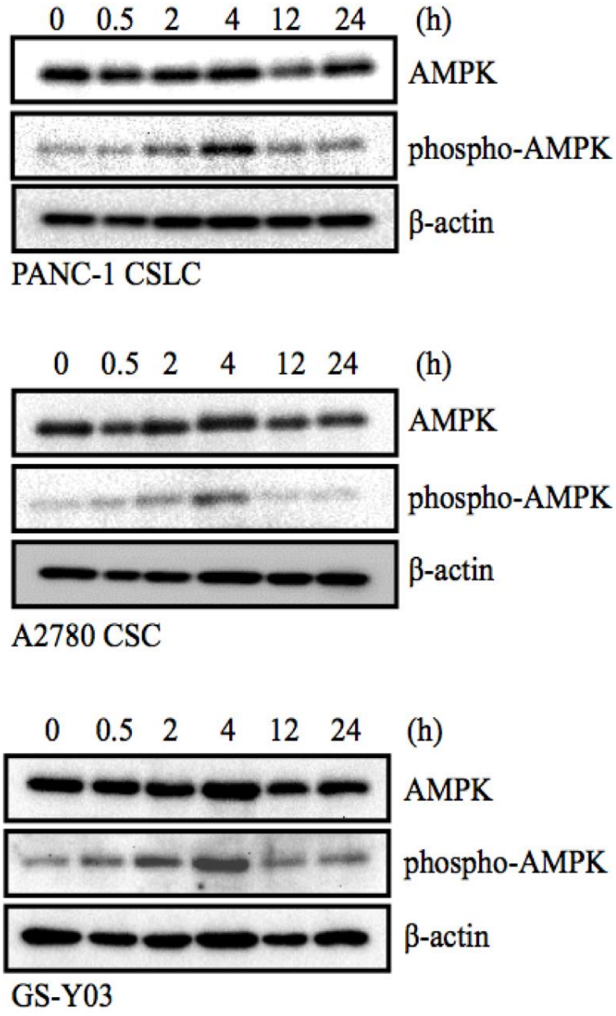

Supplemental Figure S2: The effect of WZB117 treatment on the AMPK activity of CSCs.

Cells cultured in the presence of WZB117 (10 μM for PANC-1 CSLC and 2 μM for A2780 CSC and GS-Y03) were subjected, at the indicated time points, to immunoblot analysis of phospho-AMPK.

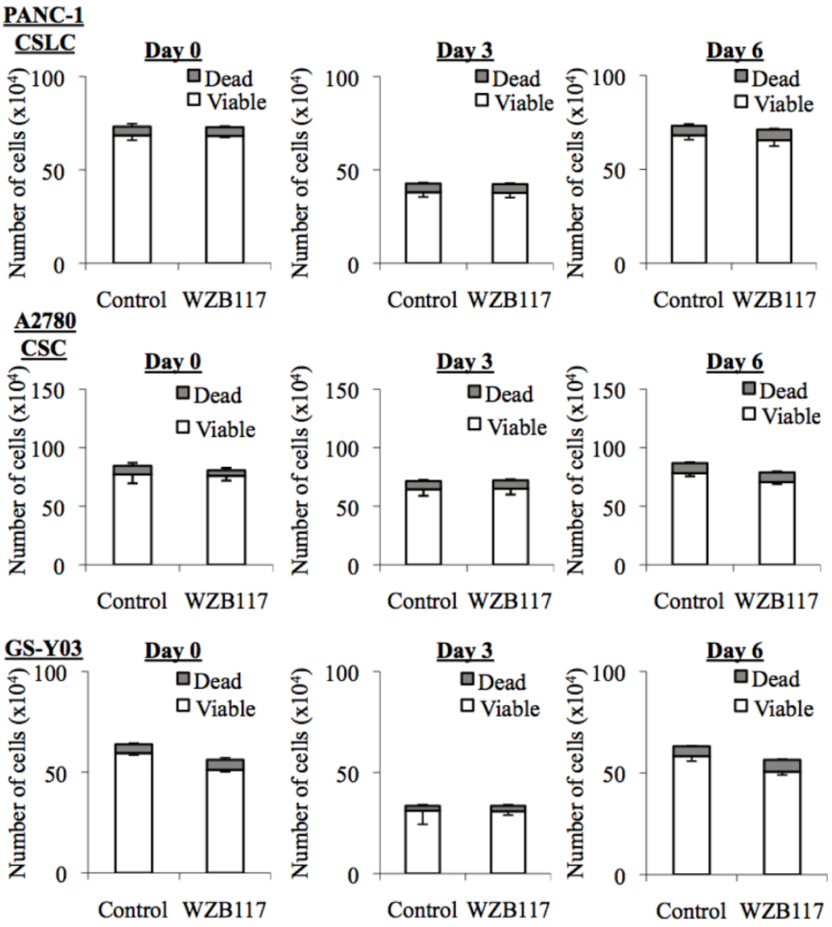

Supplemental Figure S3: The effect of WZB117 treatment on the viability of CSCs.

Cells (1 x 10^5 viable cells) were cultured in the absence (Control) or presence of WZB117 (10 μM for PANC-1 CSLC and 2 μM for A2780 CSC and GS-Y03) for 6 days. Then, the cells were either subjected to determination of the numbers of viable and dead cells by dye exclusion (Day 0) or were re-seeded (5 x 10^4 viable cells) onto collagen-coated dishes and further cultured under the monolayer stem cell culture condition in the absence of WZB117 for 3 (Day 3) and 6 (Day 6) days. The numbers of viable and dead cells were similarly determined by dye exclusion on Day 3 and Day 6, as indicated. Values represent means + SD (for dead cell numbers) and – SD (for viable cell numbers) of three independent experiments.

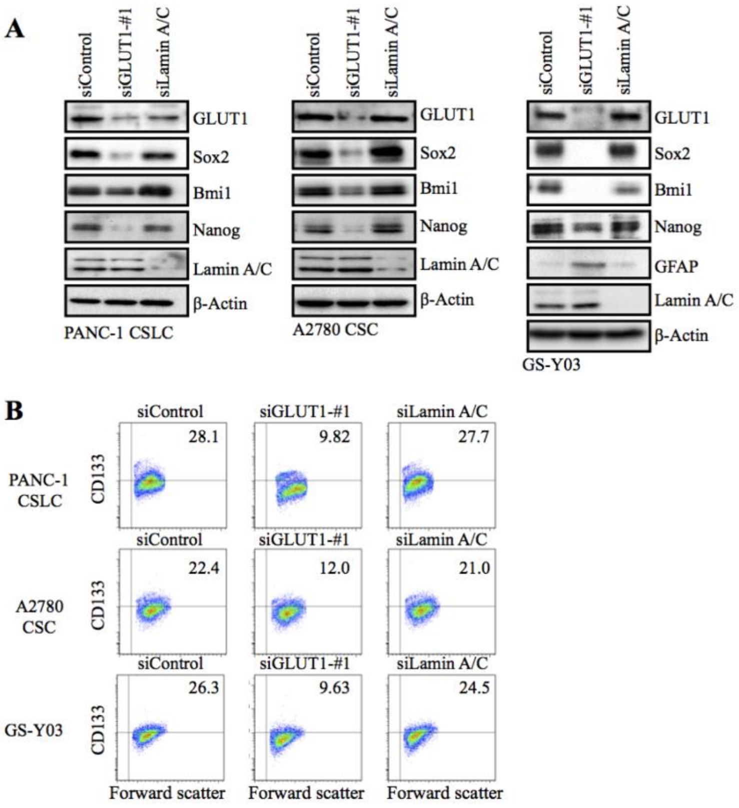

Supplemental Figure S4: The effect of siRNA-mediated GLUT1 knockdown on CSCs.

(A) Cells were transiently transfected with an siRNA against GLUT1 (siGLUT1-#1; note that siGLUT1-#3 was used in Figure 3), and as controls, with an siRNA against Lamin A/C (siLamin A/C) or with a non-targeting siRNA (siControl), as detailed in Materials and methods. The transfected cells were then subjected, 6 days after transfection for PANC-1 CSLCs and 8 days after transfection for A2780 CSCs and GS-Y03 cells, to immunoblot analyses for the expression of the indicated proteins.

(B) Cells treated as in (A) were subjected to flow cytometric analysis for the cell surface expression of CD133. Representative flow cytometric plots together with the percentages of CD133-positive cells are shown.

Note that the flow cytometric experiments in Figure 3B and Figure S4B were done at the same time. The data for siControl and siLamin A/C are therefore identical in these figures.

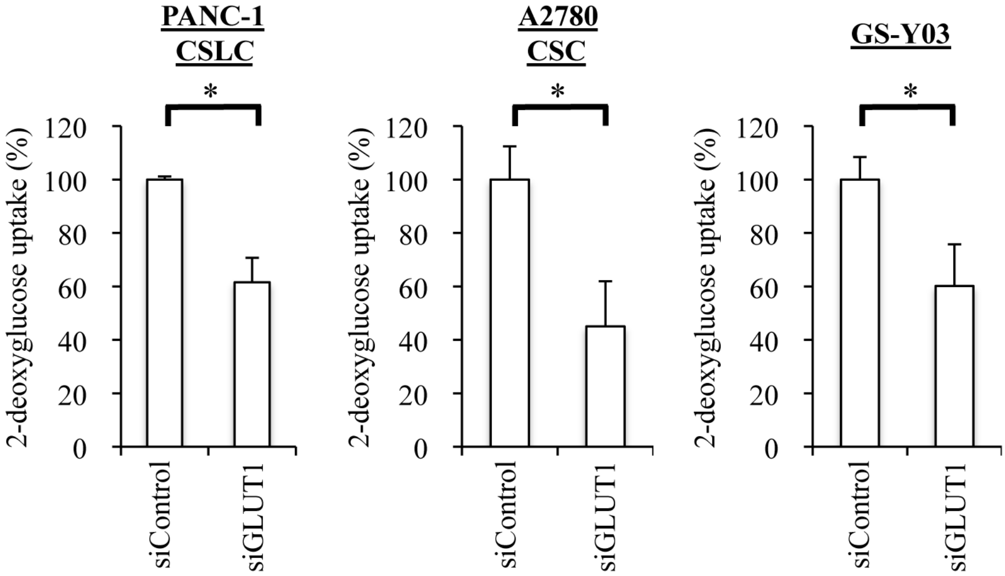

Supplemental Figure S5: The effect of siRNA-mediated GLUT1 knockdown on the glucose uptake of CSCs.

Cells were transiently transfected with an siRNA against GLUT1 (siGLUT1-#3), and as a control with a non-targeting siRNA (siControl), as detailed in Materials and methods. The transfected cells were then subjected, 6 days after transfection for PANC-1 CSLCs and 8 days after transfection for A2780 CSCs and GS-Y03 cells, to glucose-uptake assay. Values are expressed in percentage relative to those of siControl (mean values set to 100) and represent means + SD of three independent experiments. *$p<0.05$.

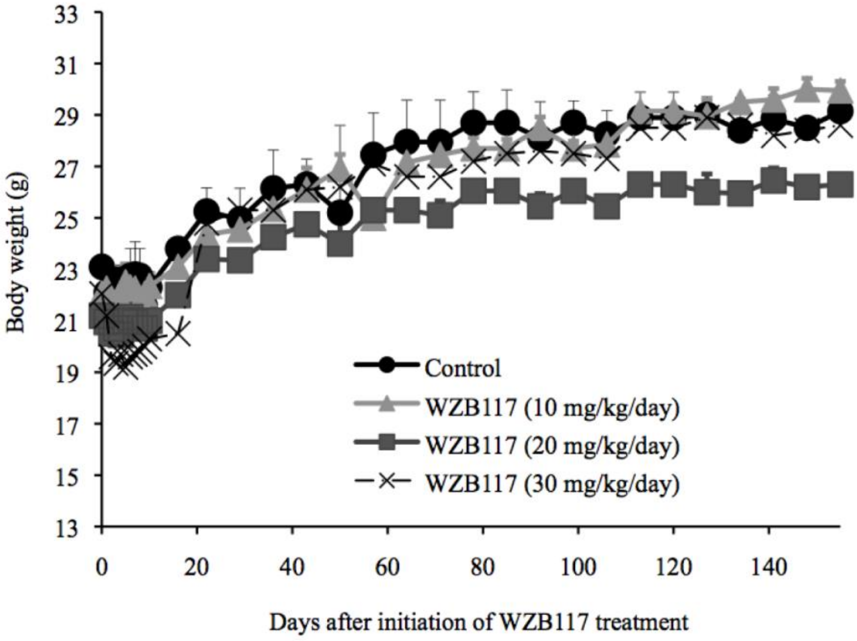

Supplemental Figure S6: The effect of systemic WZB117 administration on mouse body weight.

Four groups of mice (2 per each group) were treated with daily intraperitoneal administration of the indicated doses of WZB117 (0 = Control, 10, 20 and 30 mg/kg/day) for 10 consecutive days, and their body weight was measured at the indicated time points. Values represent means + SD of each group.

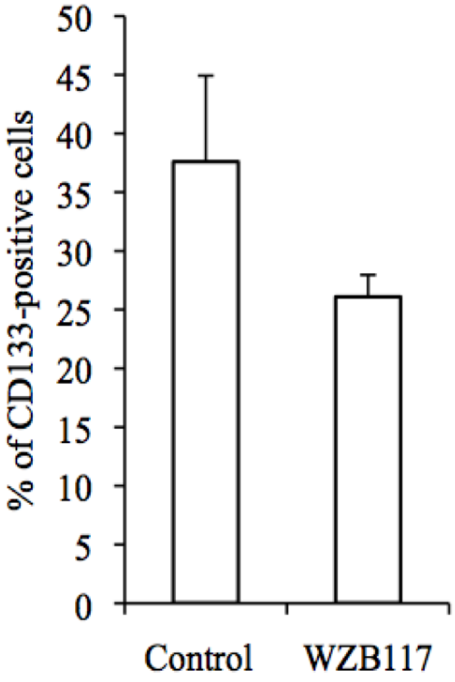

Supplemental Figure S7: The effect of systemic WZB117 administration on the proportion of CD133-positive cells in pre-established tumors.

Mice were implanted subcutaneously with PANC-1 CSLCs. Twenty days after implantation, when implanted cells formed visible tumors, the mice were randomized and received a daily intraperitoneal injection of the control vehicle or WZB117 (20 mg/kg/day) for 10 days. Then, the subcutaneous tumors (3 tumors for each treatment group) were excised and fixed after enzymatic dissociation, to subject the tumor cells to flow cytometric analysis of surface CD133 expression. Values in the graph represent means + SD of each treatment group.
